# Human Hemochromatosis Protein (HFE) Immunoperoxidase Stain Highlights Choriocarcinoma within Mixed Germ Cell Tumors

**DOI:** 10.1155/2016/5236482

**Published:** 2016-03-13

**Authors:** Jesse L. Cox, Geoffrey A. Talmon, Scott A. Koepsell

**Affiliations:** Department of Pathology and Microbiology, University of Nebraska Medical Center, Omaha, NE 68198-3135, USA

## Abstract

Identification of choriocarcinoma within a germ cell tumor can have major implications for the subsequent staging and treatment of testicular neoplasms. Immunoperoxidase staining greatly enhances the speed and sensitivity of identifying occult, though clinically significant, tumor components. In mixed germ cell tumors, staining for beta-human chorionic gonadotropin (*β*-hCG) has been historically used to assess for the presence and burden of choriocarcinoma. However, current *β*-hCG stains produce variable, intense staining of trophoblastic elements and surrounding tissues, clouding the assessment of true-positive staining. Human hemochromatosis protein (HFE) is a membrane bound mediator of iron transport expressed at high levels within placenta. Additionally, previous reports have demonstrated that choriocarcinoma cell lines express HFE, although* in vivo* expression had not been examined. To address whether HFE can stain trophoblastic elements, HFE immunohistochemistry was conducted in choriocarcinoma (*n* = 4), mixed germ cell tumors (*n* = 11), seminoma (*n* = 4), and placenta (*n* = 11). HFE consistently demonstrated cytoplasmic and membranous staining, highlighting both syncytiotrophoblasts and cytotrophoblasts within choriocarcinoma and placenta. Staining of intratumoral white blood cells was observed within seminomas and mixed germ cell tumors, corroborating prior reports stating that HFE highlights monocytes and macrophages. Taken together, HFE may serve as an alternative target from *β*-hCG for immunoperoxidase studies when highlighting choriocarcinoma.

## 1. Introduction

Identification of occult choriocarcinoma within mixed germ cell tumors has profound implications on the treatment and prognosis of affected patients [1]. Beta-human chorionic gonadotropin (*β*-hCG) has been a well-established marker of syncytiotrophoblast proliferation, both in serum and in tissue immunoperoxidase staining [2, 3]. However, current commercially available antibodies used for immunoperoxidase-based assessment of *β*-hCG expression in tissue can produce a marked amount of nonspecific staining, due in part to the autocrine/paracrine biological function of *β*-hCG, clouding the interpretation in identifying and quantifying choriocarcinoma elements within germ cell tumors.

Hemochromatosis factor (HFE) is an MHC class I-like glycoprotein, which works in concert with numerous other proteins to sense and regulate systemic iron levels (review [4]). Moreover, mutation of HFE is associated with hereditary hemochromatosis. In the placenta, syncytiotrophoblasts have been shown to express HFE [5]. Because choriocarcinoma contains syncytiotrophoblast-like cells, we hypothesized that choriocarcinoma would express HFE. Furthermore, we postulated that HFE may be used to identify occult syncytiotrophoblast-like cells within mixed germ cell tumors.

Here, we demonstrate that choriocarcinoma tumor cells, including both syncytiotrophoblasts and cytotrophoblasts, express HFE, which is detectable by immunoperoxidase staining. Moreover, we show that HFE is also expressed within cytotrophoblastic elements of cystic trophoblastic tumor and intermediate trophoblast of an exaggerated placenta site. Together, the data presented herein supports HFE as a viable marker for trophoblast progenitors.

## 2. Materials and Methods

Following the approval from the University of Nebraska Institutional Review Board, thirty (30) patient samples were identified from the University of Nebraska Medical Center (UNMC) Department of Pathology Laboratory Information System for inclusion in this study. Specimen origin and primary diagnosis are summarized in Table 1. Briefly, samples collected were predicted to stain positively or negatively with *β*-hCG. Negative staining tissues included pure seminomas without a syncytiotrophoblast component by H&E staining and mixed germ cell tumors without an identifiable choriocarcinoma component. Tissues that are expected to stain positively for *β*-hCG included placenta, hydatidiform moles, gestational trophoblastic tissues, cystic trophoblastic tissues, and mixed germ cell tumors including a readily identifiable component of choriocarcinoma and metastatic choriocarcinoma.

Tissues were fixed in formalin prior to mounting in paraffin wax, consistent with standard UNMC laboratory procedures. Initial H&E stained slides of patient samples were prepared by the UNMC Histology Department. Focal areas within specific tissue blocks were then selected for inclusion in a tissue microarray. Each tissue type was subjected to identical conditions during subsequent staining and immunohistochemical procedures. *β*-hCG (234A-18, Cell Marque, Rocklin, CA) and CD45 (760-2505, Ventana Medical Systems, Inc., Tucson AZ) staining were conducted by the UNMC Immunoperoxidase Laboratory. HFE (sc-133654, Santa Cruz Biotechnology, Inc., Dallas, TX) staining was conducted at 1 : 50 dilution by the UNMC Tissue Sciences Facility. Briefly, unstained slides prepared from paraffin embedded blocks were cleared and hydrated using xylene/alcohol on a Tissue-Tek Prisma automated stainer (Sakura Finetek USA, Inc., Torrance, CA). Next, a Leica Bond-III machine and Bond Epitope Retrieval Solution 1—pH 9.0 (Leica Biosystems Inc., Buffalo Grove, IL)—were used at 100°C for 20 minutes. After the slides were allowed to cool, they were washed and incubated with primary antibody and then washed and incubated with Envision anti-Rabbit Polymer (Dako Inc., Carpinteria, CA). Lastly, the slides were washed and developed Bond Polymer Refine Detection Kit (Leica). Counterstain was conducted with Hematoxylin. Photomicrographs were captured using iScan Coreo Au scanner and iScan Coreo 3.4.0 software (Ventana Medical Systems, Inc.).

## 3. Result and Discussion

**Figure 1:**
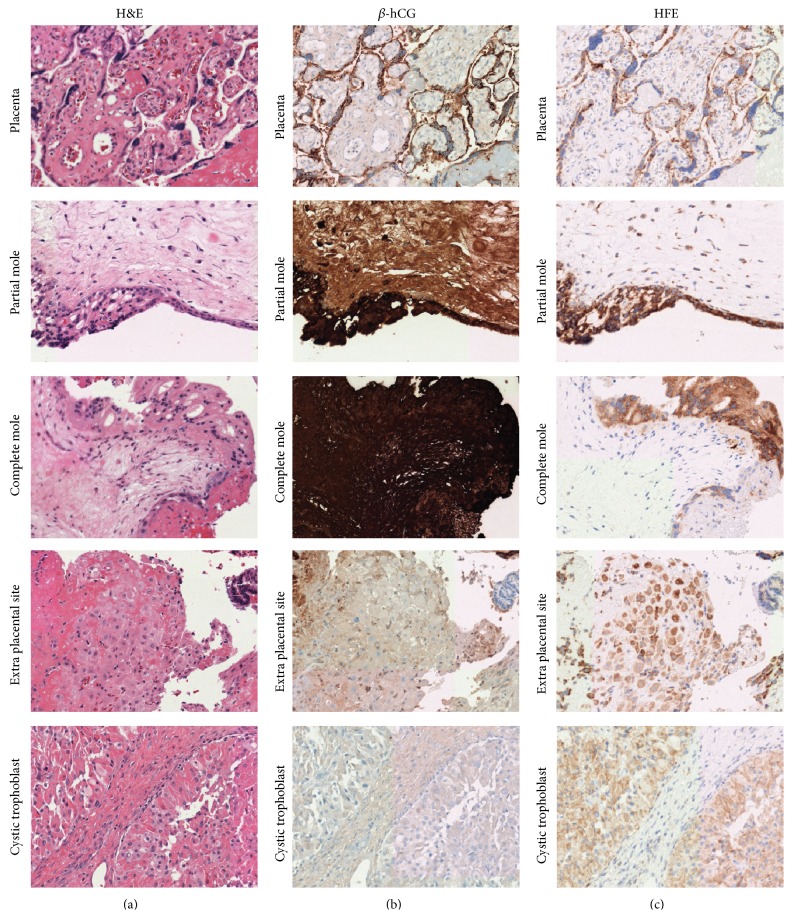
HFE staining in tissues containing syncytiotrophoblasts and/or cytotrophoblasts. Photomicrographs of tissue types containing trophoblastic elements are presented. Hematoxylin and eosin stained tissues in (a) and immunoperoxidase stained tissues for either *β*-hCG (b) or HFE (c) are shown. Photos were taken at 20x. One example of each tissue/tumor type is presented.

To confirm HFE as a viable stain of syncytiotrophoblast cells, placental tissues prepared during routine processing were examined for both *β*-hCG and HFE expression (Figure 1). As expected, the syncytiotrophoblast cells lining chorionic villi demonstrated intense expression of *β*-hCG. HFE staining demonstrated a remarkably similar pattern highlighting syncytiotrophoblast cells, corroborating previous reports identifying high levels of HFE expression in the trophoblast of placental tissue [6]. Similar *β*-hCG and HFE staining patterns were observed in complete and incomplete hydatidiform moles as well (Figure 1). We also determined whether HFE could stain other trophoblastic progenitors, in particular intermediate trophoblast and cytotrophoblast cells. For this purpose, exaggerated placental site and cystic trophoblast were examined. Interestingly, HFE was able to highlight both intermediate trophoblast elements within exaggerated placental site and cytotrophoblastic elements within cystic trophoblast (Figure 1). Five (5) additional samples of placenta and 1 additional complete mole demonstrated similar staining as their respective counterparts (data not shown).

**Figure 2:**
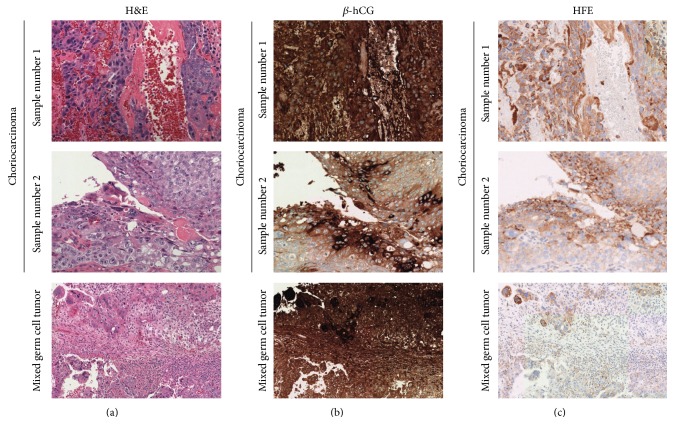
Photomicrographs of choriocarcinoma and expression of HFE and *β*-hCG. Two choriocarcinoma samples and mixed germ cell tumor with approximately 5% choriocarcinoma component are presented. Hematoxylin and eosin stained tissues in (a) and immunoperoxidase stained tissues for either *β*-hCG (b) or HFE (c) are shown. Photos were taken at 20x. One example of each tissue/tumor type is presented.

Because choriocarcinoma includes cell types synonymous with placental tissues, we suspected that choriocarcinoma cells would stain with HFE similar to *β*-hCG. Metastatic choriocarcinoma exhibited intense staining for *β*-hCG, both in syncytiotrophoblastic elements and in cytotrophoblastic elements (Figure 2). Moreover, significant variability in staining intensity was identified amongst different choriocarcinoma samples examined. Importantly, tissues examined were stained as part of a single tissue array and were subjected to the same staining conditions. Staining for HFE was also strongly positive in syncytiotrophoblastic and cytotrophoblastic elements; however, the intensity staining was reduced with HFE as compared to *β*-hCG, and staining was largely localized to the cell membrane and cytoplasm. Importantly, similar staining was seen in two other metastatic choriocarcinoma samples, including multiple foci of tumor collected at different sites, from a single patient (data not shown).

We next examined the ability of both *β*-hCG and HFE to identify trophoblastic elements within mixed germ cell tumors. For this purpose, multiple mixed germ cell tumors with a varying proportion of choriocarcinoma were examined. *β*-hCG staining exhibited marked nonspecificity, highlighting tissues not histologically compatible with choriocarcinoma (Figure 2). Conversely, HFE intensely stained cells consistent with the cytotrophoblastic elements of choriocarcinoma, while a faint blush of staining was detected in the other portions of the tumor. An additional 3 mixed germ cell tumors containing choriocarcinoma demonstrated similar staining patterns for both *β*-hCG and HFE (data not shown).

**Figure 3:**
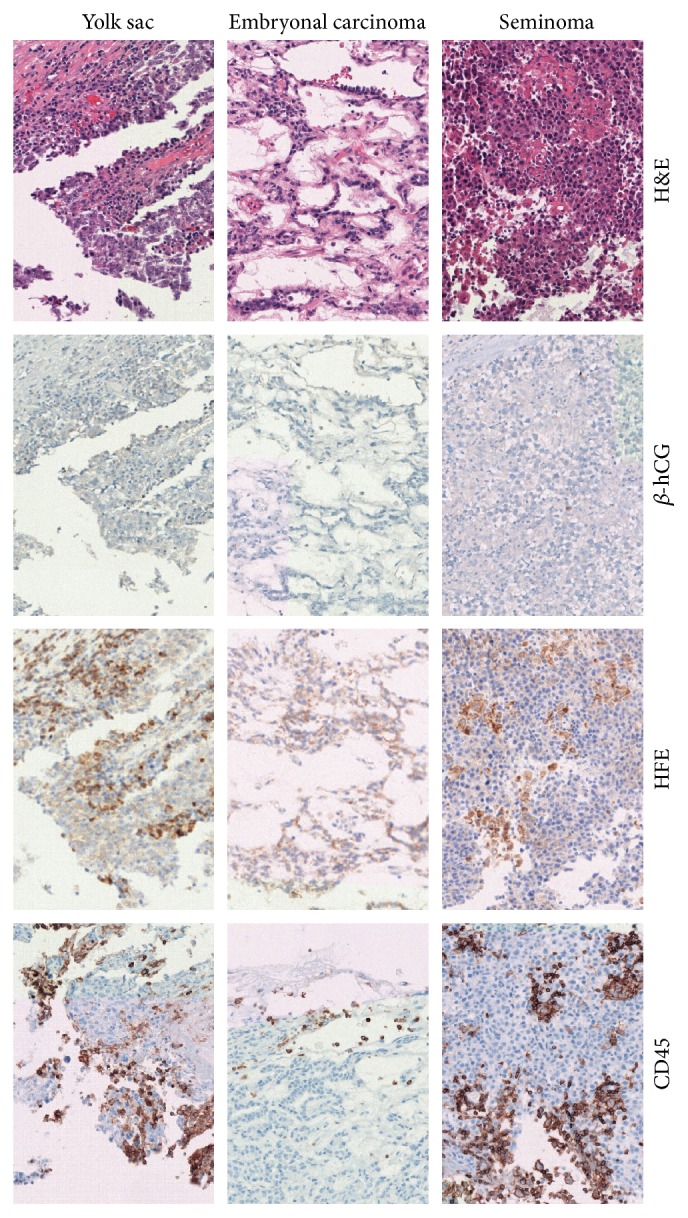
Photomicrographs of germ cell tumors without choriocarcinoma and their expression of HFE, *β*-hCG, and common leukocyte antigen, CD45. An example of a pure seminoma, a mixed germ cell tumor with a large proportion of embryonal carcinoma, and an example of a large proportion of yolk sac tumor are presented. Photos were taken at 20x.

Lastly, to determine the specificity of HFE, we examined other germ cell tumors (Figure 3). Seminoma, embryonal carcinoma, and yolk sac tumors were examined. Staining for *β*-hCG demonstrated little background staining in the germ cell tumors examined. In contrast, HFE immunohistochemical staining produced punctate staining of small cells seen within the tumor samples examined. Comparison to the corresponding H&E stained sections revealed a leukocytic infiltrate within the HFE stained areas. Previous reports have demonstrated that monocytes and granulocytes cells express HFE [7]. A CD45 stain conducted revealed a similar staining pattern as seen with HFE, consistent with prior reports in which HFE highlights inflammatory cells (Figure 3). Immunohistochemical staining of 3 additional seminoma samples as well as 5 additional mixed germ cell tumors without a choriocarcinoma component showed similar staining patterns (data not shown).

Identification of choriocarcinoma within a mixed germ cell tumor can have profound implications on patient prognosis as well as optimal treatment course [1]. Thus, enhancing our ability to detect occult choriocarcinoma can have corresponding benefit on patient outcome. Currently, *β*-hCG immunoperoxidase stains routinely used in clinical laboratories exhibit a large amount of nonspecific staining, depending on the tissue being examined. This promiscuous staining can make interpretation of occult choriocarcinoma difficult when immunoperoxidase stains are employed to help clarify routine H&E diagnosis. Interestingly, the physiologic role of *β*-hCG in the context of choriocarcinoma may explain the diffuse and oftentimes intense staining pattern observed. In this regard, *β*-hCG produced by choriocarcinoma is a hyperglycosylated form, which functions in an autocrine/paracrine manner to promote cell invasion [8]. Because of this role, *β*-hCG secreted by choriocarcinoma cells likely diffuses within the local microenvironment leading to its deposition in the surrounding interstitium. In keeping with this possibility, tissues containing trophoblastic progenitors exhibited a wide range of staining intensity. Importantly, the staining presented here was conducted using a tissue microarray in which all samples were exposed to identical staining conditions.

HFE is a regulator of iron metabolism, most notable for its association with hemochromatosis [4]. In contrast to *β*-hCG, HFE has been shown to be membrane bound and to interact with other membrane bound iron regulatory proteins, such as transferrin receptor, in human placental trophoblast cells [6]. Moreover, expression of HFE in the BeWo choriocarcinoma cell line exhibited similar localization and association as seen in placental cells [6]. Because HFE is membrane anchored, it is most likely that the relatively specific staining pattern observed from HFE within choriocarcinoma reflects the limited localization of HFE as compared to *β*-hCG. Moreover, specificity of the HFE antibody used was confirmed by conducting western blot analysis on cell lysates prepared from four lymphoma cell lines derived from patient samples and hepatocytes: two lymphoma lines and hepatocytes demonstrated high HFE expression by microarray analysis, as well as protein expression at the expected molecular weight on western blot analysis. Conversely, two lymphoma lines, which had low HFE RNA expression, had minimal staining on western blot (Koepsell et al., unpublished data). Additionally, placental tissue from multiple patients, used as gene expression controls for microarray studies, also demonstrated high gene expression of HFE by microarray analysis (Koepsell et al., unpublished data). It is important to keep in mind that other cells and tissues natively express HFE, including macrophages and monocytes [7], liver [9], and duodenum [10]. Examination of suspected metastases of choriocarcinoma to these sites not only must rely solely upon positive immunohistochemical staining of HFE, but also must take into account cellular and tissue morphology.

## 4. Conclusions

HFE expression within trophoblastic progenitors can be detected by immunohistochemical methods. Moreover, examination of HFE expression within germ cell tumors may complement and/or serve as an alternative to *β*-hCG to detect occult syncytiotrophoblastic and cytotrophoblastic elements within germ cell tumors, especially when the interpretation of *β*-hCG staining is difficult.

## Figures and Tables

**Table 1:** Demographics of patients included in this study.

1	2	Tissue source	Age	Gender	Diagnosis
	*+*	*Placenta*	*44*	*Female*	*Third trimester placenta (371 g, less than 10th percentile for gestational age) without histologic abnormality*

	+	Placenta	35	Female	Third trimester placenta (410 g, 10th–25th percentile for gestational age) without histologic abnormality

	+	Placenta	39	Female	Third trimester placenta (526 g, 10th–25th percentile for gestational age) without histologic abnormality

	+	Placenta	29	Female	Third trimester placenta (311 g, less than 10th percentile for gestational age) without histologic abnormality

	+	Placenta	25	Female	Third trimester placenta (392 g, 10th–25th percentile for gestational age) without histologic abnormality

	+	Placenta	19	Female	Third trimester placenta (447 g, 10th–25th percentile for gestational age) without histologic abnormality

	+	Products of conception	36	Female	Complete hydatidiform mole

	*+*	*Uterus, evacuation*	*46*	*Female*	*Complete hydatidiform mole*

	+	Products of conception	22	Female	Complete hydatidiform mole

	*+*	*Products of conception*	*25*	*Female*	*Features consistent with partial hydatidiform mole*

	*+*	*Uterine contents*	*26*	*Female*	*Hydropic chorionic villi with modest gestational trophoblastic proliferation*

	*+*	*Periaortic mass*	*23*	*Male*	*Cystic trophoblastic proliferation*

	**+**	**Brain, frontal lobe, right**	**24**	**Male**	**Metastatic choriocarcinoma**

	+	Lung, left upper lobe nodule	25	Male	Metastatic choriocarcinoma

*∗*	+	Brain, occipital lobe, and dura	17	Male	Metastatic mixed germ cell tumor composed of embryonal carcinoma and yolk sac tumor with focal choriocarcinoma

	**+**	**Lung, right upper lobe nodule**	**24**	**Male**	**Metastatic choriocarcinoma**

	**+**	**Testicle, left mass biopsy**	**29**	**Male**	**Mixed germ cell tumor composed of embryonal carcinoma (50%), teratoma (45%), and choriocarcinoma (5%)**

*∗*	+	Testicle, right	17	Male	Mixed germ cell tumor composed of embryonal carcinoma (70%), teratoma (20%), yolk sac tumor (5%), and choriocarcinoma (5%)

	+	Testicle, right	54	Male	Mixed germ cell tumor composed of yolk sac tumor (60%), teratoma (20%), embryonal carcinoma (10%), seminoma (5%), and choriocarcinoma (5%)

	+	Inguinal mass	28	Male	Mixed germ cell tumor composed of seminoma (40%), embryonal carcinoma (30%), and choriocarcinoma (<1%)

		**Testicle, left**	**34**	**Male**	**Classic seminoma**

		Testicle, left	65	Male	Classic seminoma

		Testicle, right	35	Male	Classic seminoma

		Testicle, left	38	Male	Classic seminoma

		Testicle, left	31	Male	Mixed germ cell tumor composed of seminoma (90%) and embryonal carcinoma (10%)

		Testicle, right	24	Male	Mixed germ cell tumor composed of teratoma (50%), embryonal carcinoma (25%), and yolk sac tumor (25%)

		Testicle, right	20	Male	Mixed germ cell tumor composed of embryonal carcinoma (>85%), yolk sac tumor (<10%), and teratoma (<10%)

		**Testicle, left**	**23**	**Male**	**Mixed germ cell tumor composed of embryonal carcinoma (99%) and teratoma (1%)**

		Testicle, right	32	Male	Mixed germ cell tumor composed of embryonal carcinoma (95%) and yolk sac tumor (5%)

		**Testicle, right**	**48**	**Male**	**Mixed germ cell tumor composed of yolk sac tumor (95%), seminoma (5%), and embryonal carcinoma (<1%)**

The patients included in this study are summarized in the table. Information includes tissue source, age, gender, and primary diagnosis. An asterisk (*∗*) in the column designated “1” denotes samples obtained from the same patient. A plus symbol (+) in the column designated “2” denotes samples with prominent HFE staining. Rows in italic font are shown in Figure 1. Rows in bold font designate tissues included in Figures 2 and 3.
